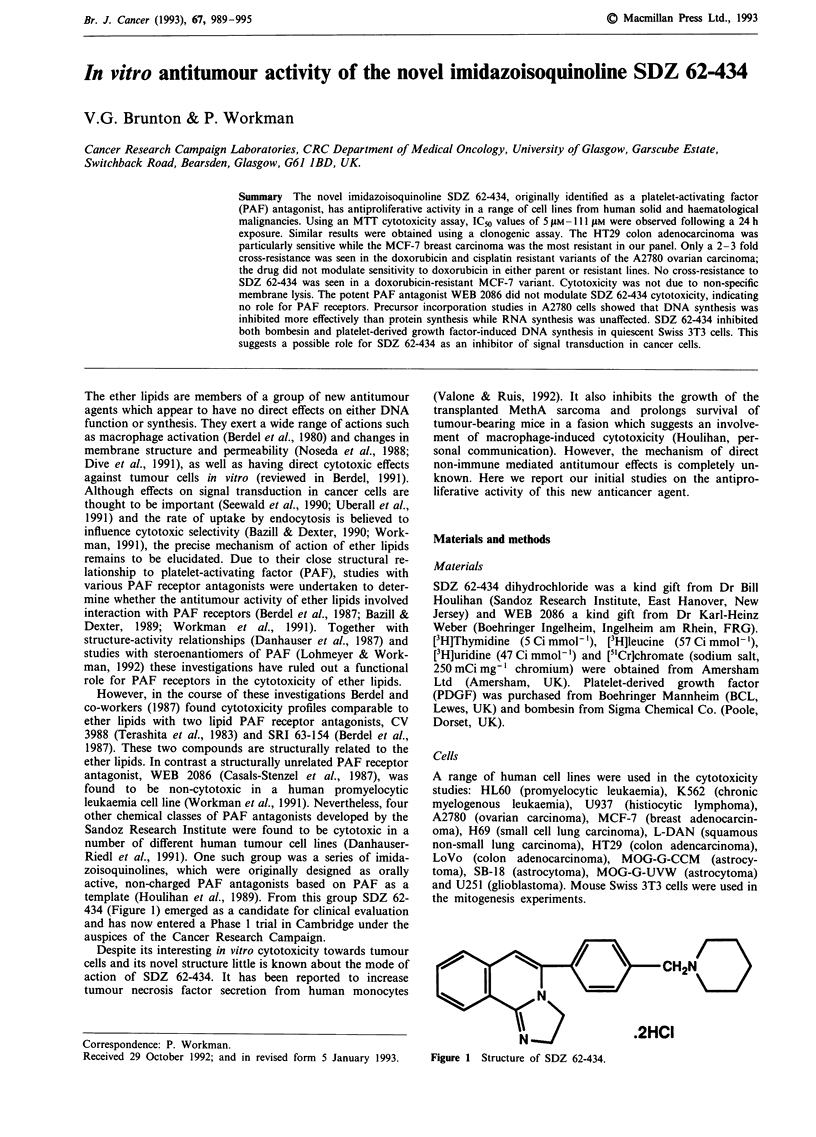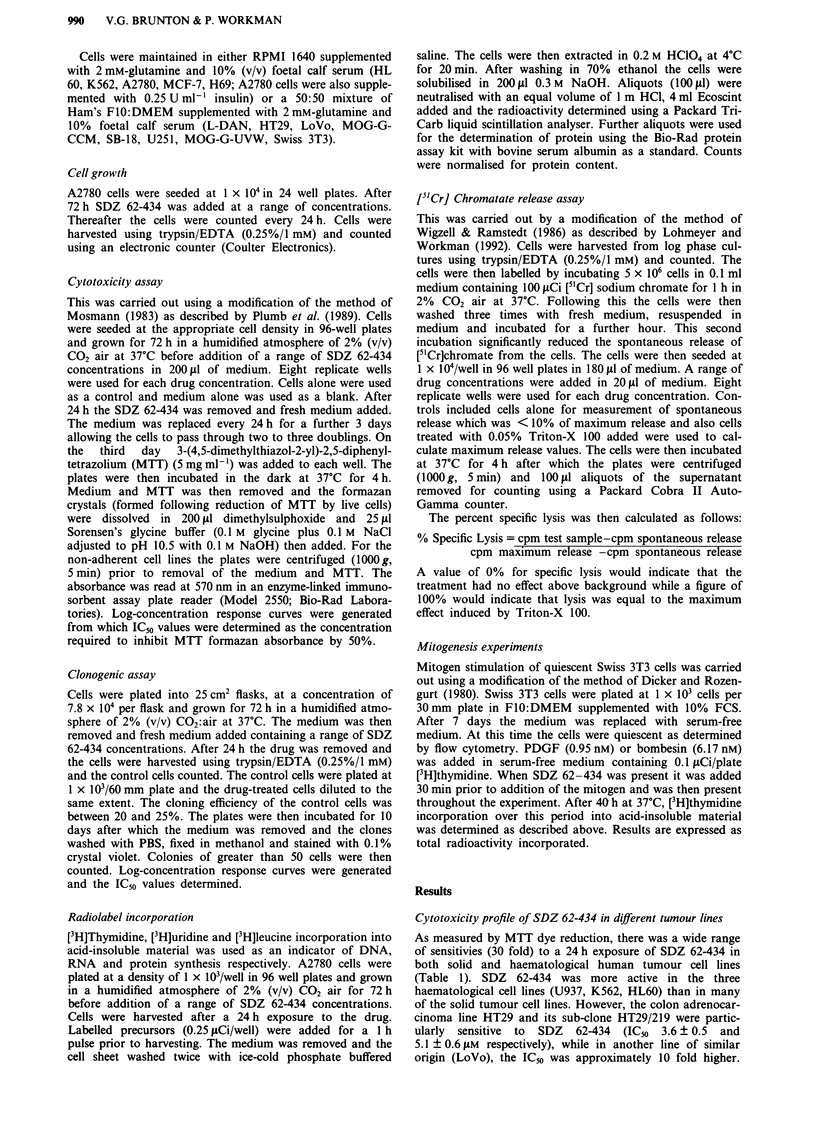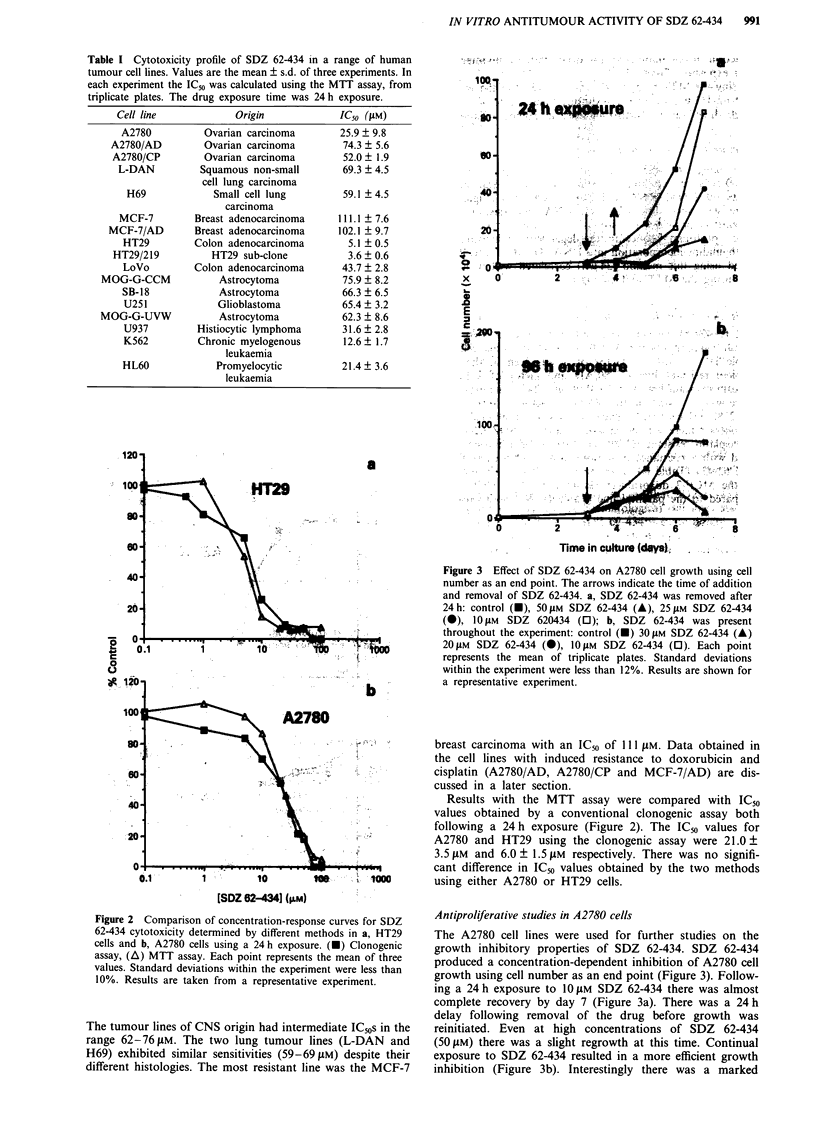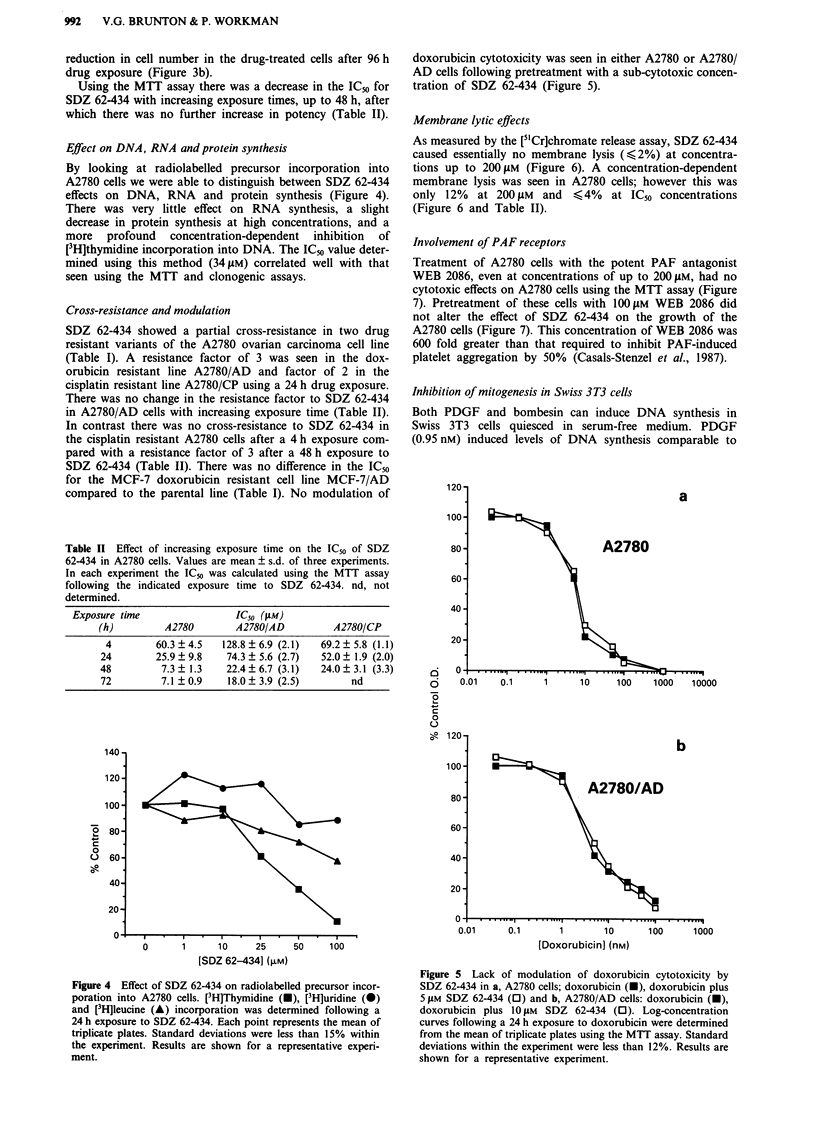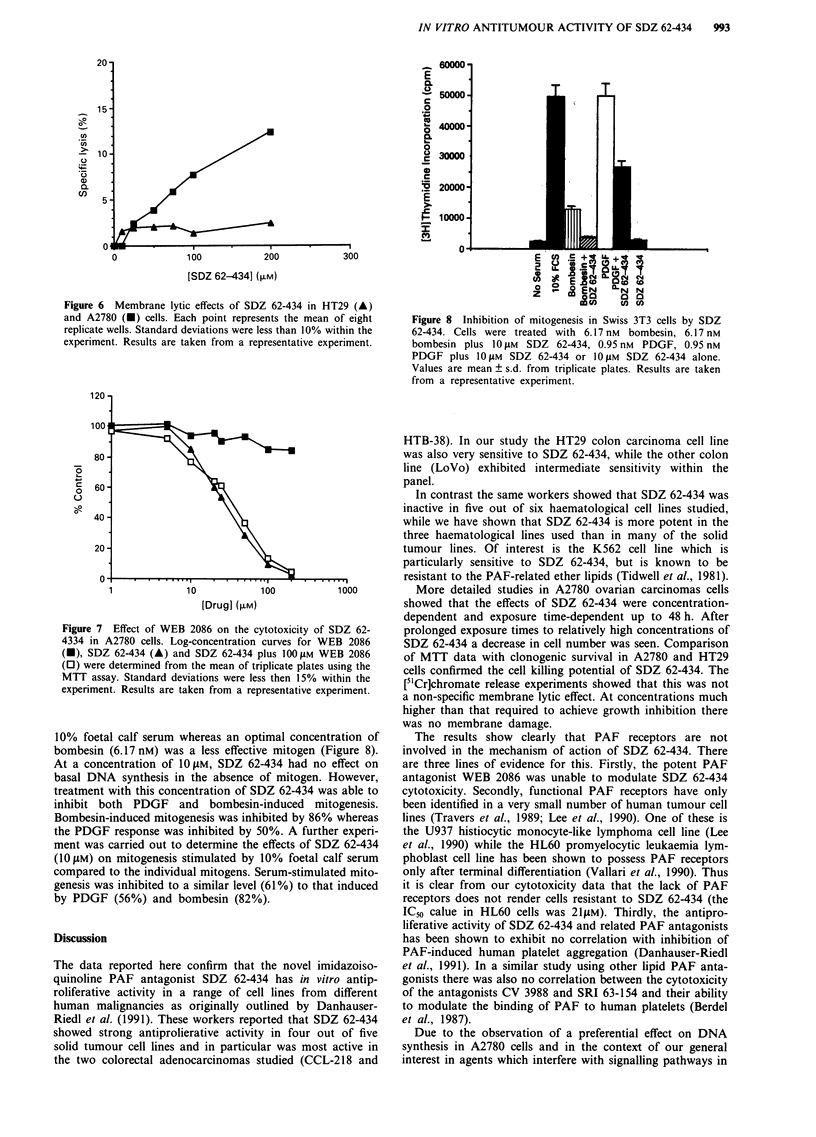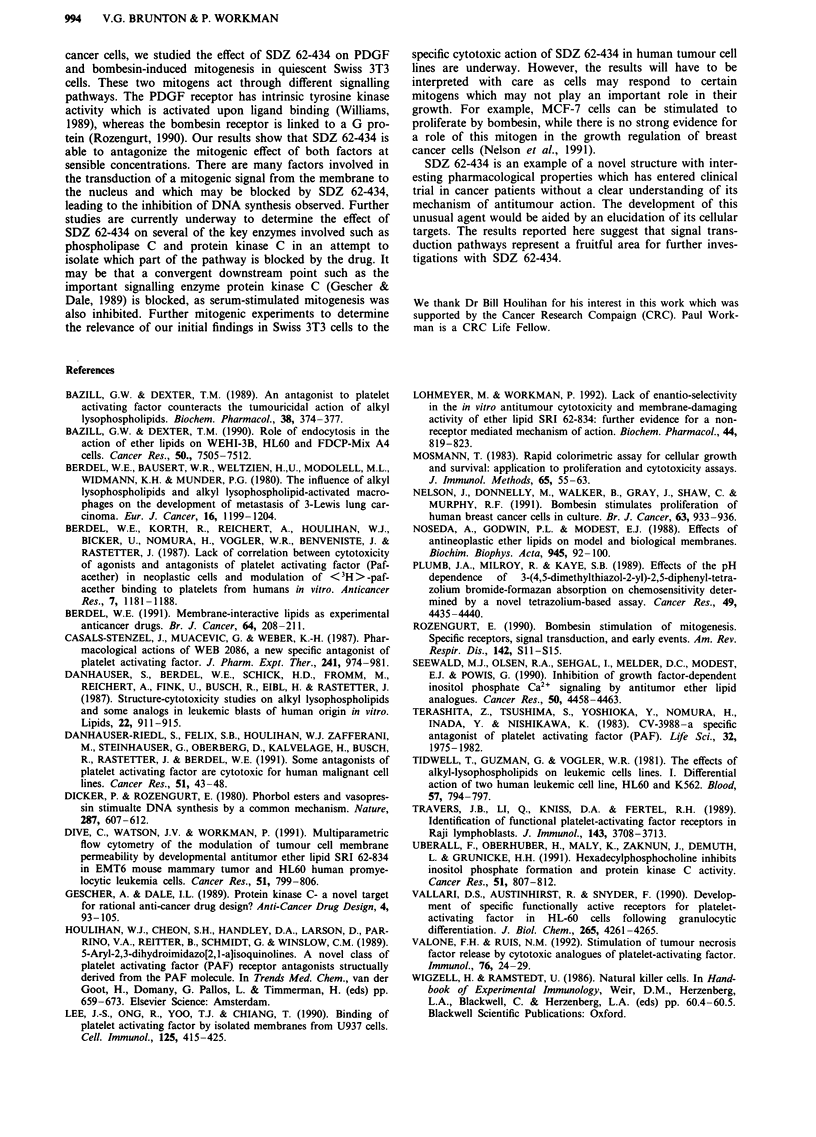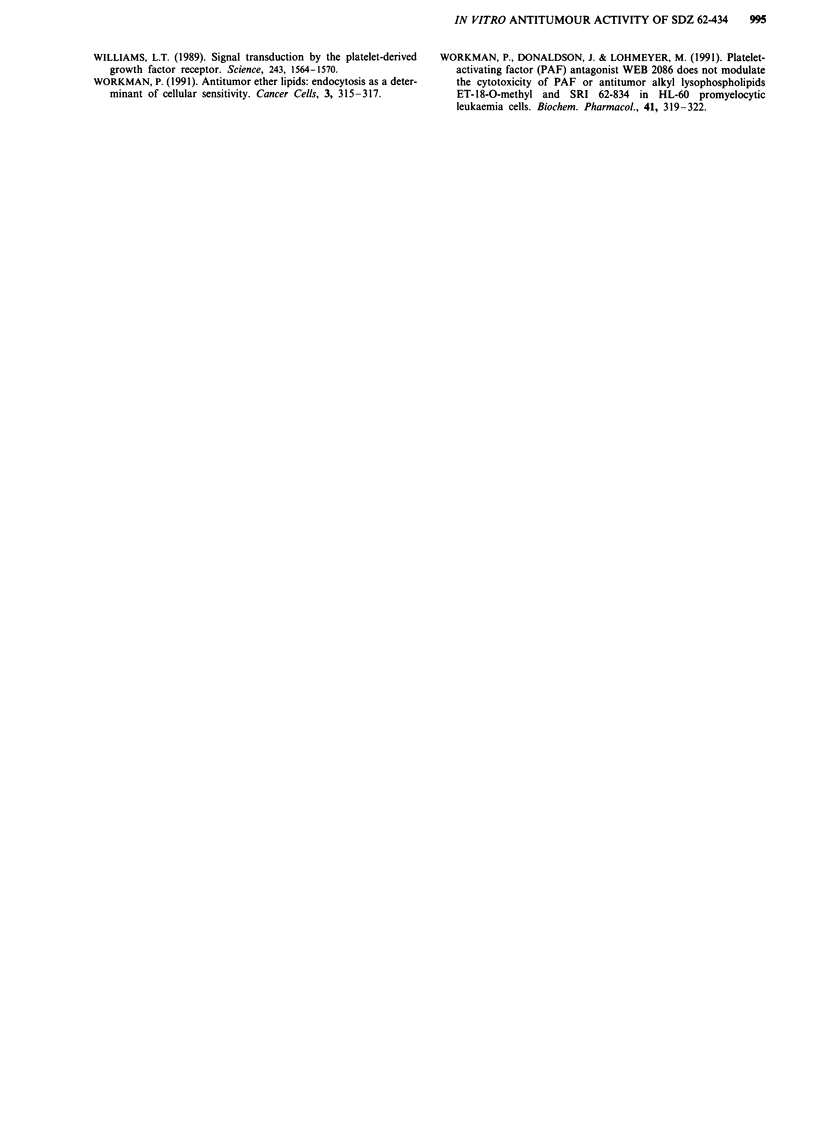# In vitro antitumour activity of the novel imidazoisoquinoline SDZ 62-434.

**DOI:** 10.1038/bjc.1993.181

**Published:** 1993-05

**Authors:** V. G. Brunton, P. Workman

**Affiliations:** CRC Department of Medical Oncology, University of Glasgow, Bearsden, UK.

## Abstract

The novel imidazoisoquinoline SDZ 62-434, originally identified as a platelet-activating factor (PAF) antagonist, has antiproliferative activity in a range of cell lines from human solid and haematological malignancies. Using an MTT cytotoxicity assay, IC50 values of 5 microM - 111 microM were observed following a 24 h exposure. Similar results were obtained using a clonogenic assay. The HT29 colon adenocarcinoma was particularly sensitive while the MCF-7 breast carcinoma was the most resistant in our panel. Only a 2-3 fold cross-resistance was seen in the doxorubicin and cisplatin resistant variants of the A2780 ovarian carcinoma; the drug did not modulate sensitivity to doxorubicin in either parent or resistant lines. No cross-resistance to SDZ 62-434 was seen in a doxorubicin-resistant MCF-7 variant. Cytotoxicity was not due to non-specific membrane lysis. The potent PAF antagonist WEB 2086 did not modulate SDZ 62-434 cytotoxicity, indicating no role for PAF receptors. Precursor incorporation studies in A2780 cells showed that DNA synthesis was inhibited more effectively than protein synthesis while RNA synthesis was unaffected. SDZ 62-434 inhibited both bombesin and platelet-derived growth factor-induced DNA synthesis in quiescent Swiss 3T3 cells. This suggests a possible role for SDZ 62-434 as an inhibitor of signal transduction in cancer cells.


Br. J. Cancer (1993), 67, 989-995 © Macmillan Press Ltd., 1993

### *In vitro* antitumour activity of the novel imidazoisoquinoline SDZ 62-434

V.G. Brunton & P. Workman

*Cancer Research Campaign Laboratories, CRC Department of Medical Oncology, University of Glasgow, Garscube Estate, Switchback Road, Bearsden, Glasgow, G61 1BD, UK.*

**Summary** The novel imidazoisoquinoline SDZ 62-434, originally identified as a platelet-activating factor (PAF) antagonist, has antiproliferative activity in a range of cell lines from human solid and haematological malignancies. Using an MTT cytotoxicity assay, $IC_{50}$ values of 5 μM–111 μM were observed following a 24 h exposure. Similar results were obtained using a clonogenic assay. The HT29 colon adenocarcinoma was particularly sensitive while the MCF-7 breast carcinoma was the most resistant in our panel. Only a 2–3 fold cross-resistance was seen in the doxorubicin and cisplatin resistant variants of the A2780 ovarian carcinoma; the drug did not modulate sensitivity to doxorubicin in either parent or resistant lines. No cross-resistance to SDZ 62-434 was seen in a doxorubicin-resistant MCF-7 variant. Cytotoxicity was not due to non-specific membrane lysis. The potent PAF antagonist WEB 2086 did not modulate SDZ 62-434 cytotoxicity, indicating no role for PAF receptors. Precursor incorporation studies in A2780 cells showed that DNA synthesis was inhibited more effectively than protein synthesis while RNA synthesis was unaffected. SDZ 62-434 inhibited both bombesin and platelet-derived growth factor-induced DNA synthesis in quiescent Swiss 3T3 cells. This suggests a possible role for SDZ 62-434 as an inhibitor of signal transduction in cancer cells.

The ether lipids are members of a group of new antitumour agents which appear to have no direct effects on either DNA function or synthesis. They exert a wide range of actions such as macrophage activation (Berdel *et al.*, 1980) and changes in membrane structure and permeability (Noseda *et al.*, 1988; Dive *et al.*, 1991), as well as having direct cytotoxic effects against tumour cells *in vitro* (reviewed in Berdel, 1991). Although effects on signal transduction in cancer cells are thought to be important (Seewald *et al.*, 1990; Uberall *et al.*, 1991) and the rate of uptake by endocytosis is believed to influence cytotoxic selectivity (Bazill & Dexter, 1990; Workman, 1991), the precise mechanism of action of ether lipids remains to be elucidated. Due to their close structural relationship to platelet-activating factor (PAF), studies with various PAF receptor antagonists were undertaken to determine whether the antitumour activity of ether lipids involved interaction with PAF receptors (Berdel *et al.*, 1987; Bazill & Dexter, 1989; Workman *et al.*, 1991). Together with structure-activity relationships (Danhauser *et al.*, 1987) and studies with steroenantiomers of PAF (Lohmeyer & Workman, 1992) these investigations have ruled out a functional role for PAF receptors in the cytotoxicity of ether lipids.

However, in the course of these investigations Berdel and co-workers (1987) found cytotoxicity profiles comparable to ether lipids with two lipid PAF receptor antagonists, CV 3988 (Terashita *et al.*, 1983) and SRI 63-154 (Berdel *et al.*, 1987). These two compounds are structurally related to the ether lipids. In contrast a structurally unrelated PAF receptor antagonist, WEB 2086 (Casals-Stenzel *et al.*, 1987), was found to be non-cytotoxic in a human promyelocytic leukaemia cell line (Workman *et al.*, 1991). Nevertheless, four other chemical classes of PAF antagonists developed by the Sandoz Research Institute were found to be cytotoxic in a number of different human tumour cell lines (Danhauser-Riedl *et al.*, 1991). One such group was a series of imidazoisoquinolines, which were originally designed as orally active, non-charged PAF antagonists based on PAF as a template (Houlihan *et al.*, 1989). From this group SDZ 62-434 (Figure 1) emerged as a candidate for clinical evaluation and has now entered a Phase 1 trial in Cambridge under the auspices of the Cancer Research Campaign.

Despite its interesting *in vitro* cytotoxicity towards tumour cells and its novel structure little is known about the mode of action of SDZ 62-434. It has been reported to increase tumour necrosis factor secretion from human monocytes (Valone & Ruis, 1992). It also inhibits the growth of the transplanted MethA sarcoma and prolongs survival of tumour-bearing mice in a fasion which suggests an involvement of macrophage-induced cytotoxicity (Houlihan, personal communication). However, the mechanism of direct non-immune mediated antitumour effects is completely unknown. Here we report our initial studies on the antiproliferative activity of this new anticancer agent.

### Materials and methods

### *Materials*

SDZ 62-434 dihydrochloride was a kind gift from Dr Bill Houlihan (Sandoz Research Institute, East Hanover, New Jersey) and WEB 2086 a kind gift from Dr Karl-Heinz Weber (Boehringer Ingelheim, Ingelheim am Rhein, FRG). [$^3$H]Thymidine (5 Ci mmol$^{-1}$), [$^3$H]leucine (57 Ci mmol$^{-1}$), [$^3$H]uridine (47 Ci mmol$^{-1}$) and [$^{51}$Cr]chromate (sodium salt, 250 mCi mg$^{-1}$ chromium) were obtained from Amersham Ltd (Amersham, UK). Platelet-derived growth factor (PDGF) was purchased from Boehringer Mannheim (BCL, Lewes, UK) and bombesin from Sigma Chemical Co. (Poole, Dorset, UK).

### *Cells*

A range of human cell lines were used in the cytotoxicity studies: HL60 (promyelocytic leukaemia), K562 (chronic myelogenous leukaemia), U937 (histiocytic lymphoma), A2780 (ovarian carcinoma), MCF-7 (breast adenocarcinoma), H69 (small cell lung carcinoma), L-DAN (squamous non-small lung carcinoma), HT29 (colon adencarcinoma), LoVo (colon adenocarcinoma), MOG-G-CCM (astrocytoma), SB-18 (astrocytoma), MOG-G-UVW (astrocytoma) and U251 (glioblastoma). Mouse Swiss 3T3 cells were used in the mitogenesis experiments.

**Figure 1** Structure of SDZ 62-434.

Correspondence: P. Workman.
Received 29 October 1992; and in revised form 5 January 1993.

Cells were maintained in either RPMI 1640 supplemented with 2 mM-glutamine and 10% (v/v) foetal calf serum (HL 60, K562, A2780, MCF-7, H69; A2780 cells were also supplemented with 0.25 U $ml^{-1}$ insulin) or a 50:50 mixture of Ham's F10:DMEM supplemented with 2 mM-glutamine and 10% foetal calf serum (L-DAN, HT29, LoVo, MOG-G-CCM, SB-18, U251, MOG-G-UVW, Swiss 3T3).

*Cell growth*

A2780 cells were seeded at $1 \times 10^4$ in 24 well plates. After 72 h SDZ 62-434 was added at a range of concentrations. Thereafter the cells were counted every 24 h. Cells were harvested using trypsin/EDTA (0.25%/1 mM) and counted using an electronic counter (Coulter Electronics).

*Cytotoxicity assay*

This was carried out using a modification of the method of Mosmann (1983) as described by Plumb *et al.* (1989). Cells were seeded at the appropriate cell density in 96-well plates and grown for 72 h in a humidified atmosphere of 2% (v/v) $CO_2$ air at 37°C before addition of a range of SDZ 62-434 concentrations in 200 µl of medium. Eight replicate wells were used for each drug concentration. Cells alone were used as a control and medium alone was used as a blank. After 24 h the SDZ 62-434 was removed and fresh medium added. The medium was replaced every 24 h for a further 3 days allowing the cells to pass through two to three doublings. On the third day 3-(4,5-dimethylthiazol-2-yl)-2,5-diphenyltetrazolium (MTT) (5 mg $ml^{-1}$) was added to each well. The plates were then incubated in the dark at 37°C for 4 h. Medium and MTT was then removed and the formazan crystals (formed following reduction of MTT by live cells) were dissolved in 200 µl dimethylsulphoxide and 25 µl Sorensen's glycine buffer (0.1 M glycine plus 0.1 M NaCl adjusted to pH 10.5 with 0.1 M NaOH) then added. For the non-adherent cell lines the plates were centrifuged (1000 *g*, 5 min) prior to removal of the medium and MTT. The absorbance was read at 570 nm in an enzyme-linked immunosorbent assay plate reader (Model 2550; Bio-Rad Laboratories). Log-concentration response curves were generated from which $IC_{50}$ values were determined as the concentration required to inhibit MTT formazan absorbance by 50%.

*Clonogenic assay*

Cells were plated into 25 $cm^2$ flasks, at a concentration of $7.8 \times 10^4$ per flask and grown for 72 h in a humidified atmosphere of 2% (v/v) $CO_2$:air at 37°C. The medium was then removed and fresh medium added containing a range of SDZ 62-434 concentrations. After 24 h the drug was removed and the cells were harvested using trypsin/EDTA (0.25%/1 mM) and the control cells counted. The control cells were plated at $1 \times 10^3$/60 mm plate and the drug-treated cells diluted to the same extent. The cloning efficiency of the control cells was between 20 and 25%. The plates were then incubated for 10 days after which the medium was removed and the clones washed with PBS, fixed in methanol and stained with 0.1% crystal violet. Colonies of greater than 50 cells were then counted. Log-concentration response curves were generated and the $IC_{50}$ values determined.

*Radiolabel incorporation*

[$^3$H]Thymidine, [$^3$H]uridine and [$^3$H]leucine incorporation into acid-insoluble material was used as an indicator of DNA, RNA and protein synthesis respectively. A2780 cells were plated at a density of $1 \times 10^3$/well in 96 well plates and grown in a humidified atmosphere of 2% (v/v) $CO_2$ air for 72 h before addition of a range of SDZ 62-434 concentrations. Cells were harvested after a 24 h exposure to the drug. Labelled precursors (0.25 µCi/well) were added for a 1 h pulse prior to harvesting. The medium was removed and the cell sheet washed twice with ice-cold phosphate buffered saline. The cells were then extracted in 0.2 M $HClO_4$ at 4°C for 20 min. After washing in 70% ethanol the cells were solubilised in 200 µl 0.3 M NaOH. Aliquots (100 µl) were neutralised with an equal volume of 1 m HCl, 4 ml Ecoscint added and the radioactivity determined using a Packard Tri-Carb liquid scintillation analyser. Further aliquots were used for the determination of protein using the Bio-Rad protein assay kit with bovine serum albumin as a standard. Counts were normalised for protein content.

*[$^{51}$Cr] Chromatate release assay*

This was carried out by a modification of the method of Wigzell & Ramstedt (1986) as described by Lohmeyer and Workman (1992). Cells were harvested from log phase cultures using trypsin/EDTA (0.25%/1 mM) and counted. The cells were then labelled by incubating $5 \times 10^6$ cells in 0.1 ml medium containing 100 µCi [$^{51}$Cr] sodium chromate for 1 h in 2% $CO_2$ air at 37°C. Following this the cells were then washed three times with fresh medium, resuspended in medium and incubated for a further hour. This second incubation significantly reduced the spontaneous release of [$^{51}$Cr]chromate from the cells. The cells were then seeded at $1 \times 10^4$/well in 96 well plates in 180 µl of medium. A range of drug concentrations were added in 20 µl of medium. Eight replicate wells were used for each drug concentration. Controls included cells alone for measurement of spontaneous release which was $<10\%$ of maximum release and also cells treated with 0.05% Triton-X 100 added were used to calculate maximum release values. The cells were then incubated at 37°C for 4 h after which the plates were centrifuged (1000 *g*, 5 min) and 100 µl aliquots of the supernatant removed for counting using a Packard Cobra II Auto-Gamma counter.

The percent specific lysis was then calculated as follows:

$$\% \text{ Specific Lysis} = \frac{\text{cpm test sample} - \text{cpm spontaneous release}}{\text{cpm maximum release} - \text{cpm spontaneous release}}$$

A value of 0% for specific lysis would indicate that the treatment had no effect above background while a figure of 100% would indicate that lysis was equal to the maximum effect induced by Triton-X 100.

*Mitogenesis experiments*

Mitogen stimulation of quiescent Swiss 3T3 cells was carried out using a modification of the method of Dicker and Rozengurt (1980). Swiss 3T3 cells were plated at $1 \times 10^3$ cells per 30 mm plate in F10:DMEM supplemented with 10% FCS. After 7 days the medium was replaced with serum-free medium. At this time the cells were quiescent as determined by flow cytometry. PDGF (0.95 nM) or bombesin (6.17 nM) was added in serum-free medium containing 0.1 µCi/plate [$^3$H]thymidine. When SDZ 62–434 was present it was added 30 min prior to addition of the mitogen and was then present throughout the experiment. After 40 h at 37°C, [$^3$H]thymidine incorporation over this period into acid-insoluble material was determined as described above. Results are expressed as total radioactivity incorporated.

### Results

*Cytotoxicity profile of SDZ 62-434 in different tumour lines*

As measured by MTT dye reduction, there was a wide range of sensitivies (30 fold) to a 24 h exposure of SDZ 62-434 in both solid and haematological human tumour cell lines (Table 1). SDZ 62-434 was more active in the three haematological cell lines (U937, K562, HL60) than in many of the solid tumour cell lines. However, the colon adrenocarcinoma line HT29 and its sub-clone HT29/219 were particularly sensitive to SDZ 62-434 ($IC_{50}$ $3.6 \pm 0.5$ and $5.1 \pm 0.6$ µM respectively), while in another line of similar origin (LoVo), the $IC_{50}$ was approximately 10 fold higher.

**Table I** Cytotoxicity profile of SDZ 62-434 in a range of human tumour cell lines. Values are the mean ± s.d. of three experiments. In each experiment the $IC_{50}$ was calculated using the MTT assay, from triplicate plates. The drug exposure time was 24 h exposure.

| *Cell line* | *Origin* | *$IC_{50}$ (μM)* |
|---|---|---|
| A2780 | Ovarian carcinoma | 25.9 ± 9.8 |
| A2780/AD | Ovarian carcinoma | 74.3 ± 5.6 |
| A2780/CP | Ovarian carcinoma | 52.0 ± 1.9 |
| L-DAN | Squamous non-small cell lung carcinoma | 69.3 ± 4.5 |
| H69 | Small cell lung carcinoma | 59.1 ± 4.5 |
| MCF-7 | Breast adenocarcinoma | 111.1 ± 7.6 |
| MCF-7/AD | Breast adenocarcinoma | 102.1 ± 9.7 |
| HT29 | Colon adenocarcinoma | 5.1 ± 0.5 |
| HT29/219 | HT29 sub-clone | 3.6 ± 0.6 |
| LoVo | Colon adenocarcinoma | 43.7 ± 2.8 |
| MOG-G-CCM | Astrocytoma | 75.9 ± 8.2 |
| SB-18 | Astrocytoma | 66.3 ± 6.5 |
| U251 | Glioblastoma | 65.4 ± 3.2 |
| MOG-G-UVW | Astrocytoma | 62.3 ± 8.6 |
| U937 | Histiocytic lymphoma | 31.6 ± 2.8 |
| K562 | Chronic myelogenous leukaemia | 12.6 ± 1.7 |
| HL60 | Promyelocytic leukaemia | 21.4 ± 3.6 |

**Figure 2** Comparison of concentration-response curves for SDZ 62-434 cytotoxicity determined by different methods in **a**, HT29 cells and **b**, A2780 cells using a 24 h exposure. (■) Clonogenic assay, (Δ) MTT assay. Each point represents the mean of three values. Standard deviations within the experiment were less than 10%. Results are taken from a representative experiment.

The tumour lines of CNS origin had intermediate $IC_{50}$s in the range 62–76 μM. The two lung tumour lines (L-DAN and H69) exhibited similar sensitivities (59–69 μM) despite their different histologies. The most resistant line was the MCF-7 breast carcinoma with an $IC_{50}$ of 111 μM. Data obtained in the cell lines with induced resistance to doxorubicin and cisplatin (A2780/AD, A2780/CP and MCF-7/AD) are discussed in a later section.

**Figure 3** Effect of SDZ 62-434 on A2780 cell growth using cell number as an end point. The arrows indicate the time of addition and removal of SDZ 62-434. **a**, SDZ 62-434 was removed after 24 h: control (■), 50 μM SDZ 62-434 (▲), 25 μM SDZ 62-434 (●), 10 μM SDZ 620434 (□); **b**, SDZ 62-434 was present throughout the experiment: control (■) 30 μM SDZ 62-434 (▲) 20 μM SDZ 62-434 (●), 10 μM SDZ 62-434 (□). Each point represents the mean of triplicate plates. Standard deviations within the experiment were less than 12%. Results are shown for a representative experiment.

Results with the MTT assay were compared with $IC_{50}$ values obtained by a conventional clonogenic assay both following a 24 h exposure (Figure 2). The $IC_{50}$ values for A2780 and HT29 using the clonogenic assay were 21.0 ± 3.5 μM and 6.0 ± 1.5 μM respectively. There was no significant difference in $IC_{50}$ values obtained by the two methods using either A2780 or HT29 cells.

### *Antiproliferative studies in A2780 cells*

The A2780 cell lines were used for further studies on the growth inhibitory properties of SDZ 62-434. SDZ 62-434 produced a concentration-dependent inhibition of A2780 cell growth using cell number as an end point (Figure 3). Following a 24 h exposure to 10 μM SDZ 62-434 there was almost complete recovery by day 7 (Figure 3a). There was a 24 h delay following removal of the drug before growth was reinitiated. Even at high concentrations of SDZ 62-434 (50 μM) there was a slight regrowth at this time. Continual exposure to SDZ 62-434 resulted in a more efficient growth inhibition (Figure 3b). Interestingly there was a marked reduction in cell number in the drug-treated cells after 96 h drug exposure (Figure 3b).

Using the MTT assay there was a decrease in the $IC_{50}$ for SDZ 62-434 with increasing exposure times, up to 48 h, after which there was no further increase in potency (Table II).

*Effect on DNA, RNA and protein synthesis*

By looking at radiolabelled precursor incorporation into A2780 cells we were able to distinguish between SDZ 62-434 effects on DNA, RNA and protein synthesis (Figure 4). There was very little effect on RNA synthesis, a slight decrease in protein synthesis at high concentrations, and a more profound concentration-dependent inhibition of $[^3H]$thymidine incorporation into DNA. The $IC_{50}$ value determined using this method (34 μM) correlated well with that seen using the MTT and clonogenic assays.

*Cross-resistance and modulation*

SDZ 62-434 showed a partial cross-resistance in two drug resistant variants of the A2780 ovarian carcinoma cell line (Table I). A resistance factor of 3 was seen in the doxorubicin resistant line A2780/AD and factor of 2 in the cisplatin resistant line A2780/CP using a 24 h drug exposure. There was no change in the resistance factor to SDZ 62-434 in A2780/AD cells with increasing exposure time (Table II). In contrast there was no cross-resistance to SDZ 62-434 in the cisplatin resistant A2780 cells after a 4 h exposure compared with a resistance factor of 3 after a 48 h exposure to SDZ 62-434 (Table II). There was no difference in the $IC_{50}$ for the MCF-7 doxorubicin resistant cell line MCF-7/AD compared to the parental line (Table I). No modulation of doxorubicin cytotoxicity was seen in either A2780 or A2780/AD cells following pretreatment with a sub-cytotoxic concentration of SDZ 62-434 (Figure 5).

*Membrane lytic effects*

As measured by the $[^{51}Cr]$chromate release assay, SDZ 62-434 caused essentially no membrane lysis (≤2%) at concentrations up to 200 μM (Figure 6). A concentration-dependent membrane lysis was seen in A2780 cells; however this was only 12% at 200 μM and ≤4% at $IC_{50}$ concentrations (Figure 6 and Table II).

*Involvement of PAF receptors*

Treatment of A2780 cells with the potent PAF antagonist WEB 2086, even at concentrations of up to 200 μM, had no cytotoxic effects on A2780 cells using the MTT assay (Figure 7). Pretreatment of these cells with 100 μM WEB 2086 did not alter the effect of SDZ 62-434 on the growth of the A2780 cells (Figure 7). This concentration of WEB 2086 was 600 fold greater than that required to inhibit PAF-induced platelet aggregation by 50% (Casals-Stenzel *et al.*, 1987).

*Inhibition of mitogenesis in Swiss 3T3 cells*

Both PDGF and bombesin can induce DNA synthesis in Swiss 3T3 cells quiesced in serum-free medium. PDGF (0.95 nM) induced levels of DNA synthesis comparable to

**Table II** Effect of increasing exposure time on the $IC_{50}$ of SDZ 62-434 in A2780 cells. Values are mean ± s.d. of three experiments. In each experiment the $IC_{50}$ was calculated using the MTT assay following the indicated exposure time to SDZ 62-434. nd, not determined.

| *Exposure time (h)* | *A2780* | *$IC_{50}$ (μM)* *A2780/AD* | *A2780/CP* |
|---|---|---|---|
| 4 | 60.3 ± 4.5 | 128.8 ± 6.9 (2.1) | 69.2 ± 5.8 (1.1) |
| 24 | 25.9 ± 9.8 | 74.3 ± 5.6 (2.7) | 52.0 ± 1.9 (2.0) |
| 48 | 7.3 ± 1.3 | 22.4 ± 6.7 (3.1) | 24.0 ± 3.1 (3.3) |
| 72 | 7.1 ± 0.9 | 18.0 ± 3.9 (2.5) | nd |

**Figure 4** Effect of SDZ 62-434 on radiolabelled precursor incorporation into A2780 cells. $[^3H]$Thymidine (■), $[^3H]$uridine (●) and $[^3H]$leucine (▲) incorporation was determined following a 24 h exposure to SDZ 62-434. Each point represents the mean of triplicate plates. Standard deviations were less than 15% within the experiment. Results are shown for a representative experiment.

**Figure 5** Lack of modulation of doxorubicin cytotoxicity by SDZ 62-434 in **a**, A2780 cells; doxorubicin (■), doxorubicin plus 5 μM SDZ 62-434 (□) and **b**, A2780/AD cells: doxorubicin (■), doxorubicin plus 10 μM SDZ 62-434 (□). Log-concentration curves following a 24 h exposure to doxorubicin were determined from the mean of triplicate plates using the MTT assay. Standard deviations within the experiment were less than 12%. Results are shown for a representative experiment.

**Figure 6** Membrane lytic effects of SDZ 62-434 in HT29 (▲) and A2780 (■) cells. Each point represents the mean of eight replicate wells. Standard deviations were less than 10% within the experiment. Results are taken from a representative experiment.

**Figure 7** Effect of WEB 2086 on the cytotoxicity of SDZ 62-4334 in A2780 cells. Log-concentration curves for WEB 2086 (■), SDZ 62-434 (▲) and SDZ 62-434 plus 100 μM WEB 2086 (□) were determined from the mean of triplicate plates using the MTT assay. Standard deviations were less then 15% within the experiment. Results are taken from a representative experiment.

**Figure 8** Inhibition of mitogenesis in Swiss 3T3 cells by SDZ 62-434. Cells were treated with 6.17 nM bombesin, 6.17 nM bombesin plus 10 μM SDZ 62-434, 0.95 nM PDGF, 0.95 nM PDGF plus 10 μM SDZ 62-434 or 10 μM SDZ 62-434 alone. Values are mean ± s.d. from triplicate plates. Results are taken from a representative experiment.

10% foetal calf serum whereas an optimal concentration of bombesin (6.17 nM) was a less effective mitogen (Figure 8). At a concentration of 10 μM, SDZ 62-434 had no effect on basal DNA synthesis in the absence of mitogen. However, treatment with this concentration of SDZ 62-434 was able to inhibit both PDGF and bombesin-induced mitogenesis. Bombesin-induced mitogenesis was inhibited by 86% whereas the PDGF response was inhibited by 50%. A further experiment was carried out to determine the effects of SDZ 62-434 (10 μM) on mitogenesis stimulated by 10% foetal calf serum compared to the individual mitogens. Serum-stimulated mitogenesis was inhibited to a similar level (61%) to that induced by PDGF (56%) and bombesin (82%).

### Discussion

The data reported here confirm that the novel imidazoisoquinoline PAF antagonist SDZ 62-434 has *in vitro* antiproliferative activity in a range of cell lines from different human malignancies as originally outlined by Danhauser-Riedl *et al.* (1991). These workers reported that SDZ 62-434 showed strong antiprolierative activity in four out of five solid tumour cell lines and in particular was most active in the two colorectal adenocarcinomas studied (CCL-218 and HTB-38). In our study the HT29 colon carcinoma cell line was also very sensitive to SDZ 62-434, while the other colon line (LoVo) exhibited intermediate sensitivity within the panel.

In contrast the same workers showed that SDZ 62-434 was inactive in five out of six haematological cell lines studied, while we have shown that SDZ 62-434 is more potent in the three haematological lines used than in many of the solid tumour lines. Of interest is the K562 cell line which is particularly sensitive to SDZ 62-434, but is known to be resistant to the PAF-related ether lipids (Tidwell *et al.*, 1981).

More detailed studies in A2780 ovarian carcinomas cells showed that the effects of SDZ 62-434 were concentration-dependent and exposure time-dependent up to 48 h. After prolonged exposure times to relatively high concentrations of SDZ 62-434 a decrease in cell number was seen. Comparison of MTT data with clonogenic survival in A2780 and HT29 cells confirmed the cell killing potential of SDZ 62-434. The [$^{51}$Cr]chromate release experiments showed that this was not a non-specific membrane lytic effect. At concentrations much higher than that required to achieve growth inhibition there was no membrane damage.

The results show clearly that PAF receptors are not involved in the mechanism of action of SDZ 62-434. There are three lines of evidence for this. Firstly, the potent PAF antagonist WEB 2086 was unable to modulate SDZ 62-434 cytotoxicity. Secondly, functional PAF receptors have only been identified in a very small number of human tumour cell lines (Travers *et al.*, 1989; Lee *et al.*, 1990). One of these is the U937 histiocytic monocyte-like lymphoma cell line (Lee *et al.*, 1990) while the HL60 promyelocytic leukaemia lymphoblast cell line has been shown to possess PAF receptors only after terminal differentiation (Vallari *et al.*, 1990). Thus it is clear from our cytotoxicity data that the lack of PAF receptors does not render cells resistant to SDZ 62-434 (the $IC_{50}$ calue in HL60 cells was 21μM). Thirdly, the antiproliferative activity of SDZ 62-434 and related PAF antagonists has been shown to exhibit no correlation with inhibition of PAF-induced human platelet aggregation (Danhauser-Riedl *et al.*, 1991). In a similar study using other lipid PAF antagonists there was also no correlation between the cytotoxicity of the antagonists CV 3988 and SRI 63-154 and their ability to modulate the binding of PAF to human platelets (Berdel *et al.*, 1987).

Due to the observation of a preferential effect on DNA synthesis in A2780 cells and in the context of our general interest in agents which interfere with signalling pathways in cancer cells, we studied the effect of SDZ 62-434 on PDGF and bombesin-induced mitogenesis in quiescent Swiss 3T3 cells. These two mitogens act through different signalling pathways. The PDGF receptor has intrinsic tyrosine kinase activity which is activated upon ligand binding (Williams, 1989), whereas the bombesin receptor is linked to a G protein (Rozengurt, 1990). Our results show that SDZ 62-434 is able to antagonize the mitogenic effect of both factors at sensible concentrations. There are many factors involved in the transduction of a mitogenic signal from the membrane to the nucleus and which may be blocked by SDZ 62-434, leading to the inhibition of DNA synthesis observed. Further studies are currently underway to determine the effect of SDZ 62-434 on several of the key enzymes involved such as phospholipase C and protein kinase C in an attempt to isolate which part of the pathway is blocked by the drug. It may be that a convergent downstream point such as the important signalling enzyme protein kinase C (Gescher & Dale, 1989) is blocked, as serum-stimulated mitogenesis was also inhibited. Further mitogenic experiments to determine the relevance of our initial findings in Swiss 3T3 cells to the specific cytotoxic action of SDZ 62-434 in human tumour cell lines are underway. However, the results will have to be interpreted with care as cells may respond to certain mitogens which may not play an important role in their growth. For example, MCF-7 cells can be stimulated to proliferate by bombesin, while there is no strong evidence for a role of this mitogen in the growth regulation of breast cancer cells (Nelson *et al.*, 1991).

SDZ 62-434 is an example of a novel structure with interesting pharmacological properties which has entered clinical trial in cancer patients without a clear understanding of its mechanism of antitumour action. The development of this unusual agent would be aided by an elucidation of its cellular targets. The results reported here suggest that signal transduction pathways represent a fruitful area for further investigations with SDZ 62-434.

We thank Dr Bill Houlihan for his interest in this work which was supported by the Cancer Research Compaign (CRC). Paul Workman is a CRC Life Fellow.

### References

BAZILL, G.W. & DEXTER, T.M. (1989). An antagonist to platelet activating factor counteracts the tumouricidal action of alkyl lysophospholipids. *Biochem. Pharmacol.*, **38**, 374–377.

BAZILL, G.W. & DEXTER, T.M. (1990). Role of endocytosis in the action of ether lipids on WEHI-3B, HL60 and FDCP-Mix A4 cells. *Cancer Res.*, **50.**, 7505–7512.

BERDEL, W.E., BAUSERT, W.R., WELTZIEN, H.,U., MODOLELL, M.L., WIDMANN, K.H. & MUNDER, P.G. (1980). The influence of alkyl lysophospholipids and alkyl lysophospholipid-activated macrophages on the development of metastasis of 3-Lewis lung carcinoma. *Eur. J. Cancer*, **16**, 1199–1204.

BERDEL, W.E., KORTH, R., REICHERT, A., HOULIHAN, W.J., BICKER, U., NOMURA, H., VOGLER, W.R., BENVENISTE, J. & RASTETTER, J. (1987). Lack of correlation between cytotoxicity of agonists and antagonists of platelet activating factor (Paf-acether) in neoplastic cells and modulation of <$^3$H>-paf-acether binding to platelets from humans *in vitro. Anticancer Res.*, **7**, 1181–1188.

BERDEL, W.E. (1991). Membrane-interactive lipids as experimental anticancer drugs. *Br. J. Cancer*, **64**, 208–211.

CASALS-STENZEL, J., MUACEVIC, G. & WEBER, K.-H. (1987). Pharmacological actions of WEB 2086, a new specific antagonist of platelet activating factor. *J. Pharm. Expt. Ther.*, **241**, 974–981.

DANHAUSER, S., BERDEL, W.E., SCHICK, H.D., FROMM, M., REICHERT, A., FINK, U., BUSCH, R., EIBL, H. & RASTETTER, J. (1987). Structure-cytotoxicity studies on alkyl lysophospholipids and some analogs in leukemic blasts of human origin *in vitro*. Lipids, **22**, 911–915.

DANHAUSER-RIEDL, S., FELIX, S.B., HOULIHAN, W.J. ZAFFERANI, M., STEINHAUSER, G., OBERBERG, D., KALVELAGE, H., BUSCH, R., RASTETTER, J. & BERDEL, W.E. (1991). Some antagonists of platelet activating factor are cytotoxic for human malignant cell lines. *Cancer Res.*, **51**, 43–48.

DICKER, P. & ROZENGURT, E. (1980). Phorbol esters and vasopressin stimualte DNA synthesis by a common mechanism. *Nature*, **287**, 607–612.

DIVE, C., WATSON, J.V. & WORKMAN, P. (1991). Multiparametric flow cytometry of the modulation of tumour cell membrane permeability by developmental antitumor ether lipid SRI 62-834 in EMT6 mouse mammary tumor and HL60 human promyelocytic leukemia cells. *Cancer Res.*, **51**, 799–806.

GESCHER, A. & DALE, I.L. (1989). Protein kinase C- a novel target for rational anti-cancer drug design? *Anti-Cancer Drug Design*, **4**, 93–105.

HOULIHAN, W.J., CHEON, S.H., HANDLEY, D.A., LARSON, D., PARRINO, V.A., REITTER, B., SCHMIDT, G. & WINSLOW, C.M. (1989). 5-Aryl-2,3-dihydroimidazo[2,1-a]isoquinolines. A novel class of platelet activating factor (PAF) receptor antagonists structually derived from the PAF molecule. In *Trends Med. Chem.*, van der Goot, H., Domany, G. Pallos, L. & Timmerman, H. (eds) pp. 659–673. Elsevier Science: Amsterdam.

LEE, J.-S., ONG, R., YOO, T.J. & CHIANG, T. (1990). Binding of platelet activating factor by isolated membranes from U937 cells. *Cell. Immunol.*, **125**, 415–425.

LOHMEYER, M. & WORKMAN, P. 1992). Lack of enantio-selectivity in the *in vitro* antitumour cytotoxicity and membrane-damaging activity of ether lipid SRI 62-834: further evidence for a non-receptor mediated mechanism of action. *Biochem. Pharmacol.*, **44**, 819–823.

MOSMANN, T. (1983). Rapid colorimetric assay for cellular growth and survival: application to proliferation and cytotoxicity assays. *J. Immunol. Methods*, **65**, 55–63.

NELSON, J., DONNELLY, M., WALKER, B., GRAY, J., SHAW, C. & MURPHY, R.F. (1991). Bombesin stimulates proliferation of human breast cancer cells in culture. *Br. J. Cancer*, **63**, 933–936.

NOSEDA, A., GODWIN, P.L. & MODEST, E.J. (1988). Effects of antineoplastic ether lipids on model and biological membranes. *Biochim. Biophys. Acta*, **945**, 92–100.

PLUMB, J.A., MILROY, R. & KAYE, S.B. (1989). Effects of the pH dependence of 3-(4,5-dimethylthiazol-2-yl)-2,5-diphenyl-tetrazolium bromide-formazan absorption on chemosensitivity determined by a novel tetrazolium-based assay. *Cancer Res.*, **49**, 4435–4440.

ROZENGURT, E. (1990). Bombesin stimulation of mitogenesis. Specific receptors, signal transduction, and early events. *Am. Rev. Respir. Dis.*, **142**, S11–S15.

SEEWALD, M.J., OLSEN, R.A., SEHGAL, I., MELDER, D.C., MODEST, E.J. & POWIS, G. (1990). Inhibition of growth factor-dependent inositol phosphate $Ca^{2+}$ signaling by antitumor ether lipid analogues. *Cancer Res.*, **50**, 4458–4463.

TERASHITA, Z., TSUSHIMA, S., YOSHIOKA, Y., NOMURA, H., INADA, Y. & NISHIKAWA, K. (1983). CV-3988-a specific antagonist of platelet activating factor (PAF). *Life Sci.*, **32**, 1975–1982.

TIDWELL, T., GUZMAN, G. & VOGLER, W.R. (1981). The effects of alkyl-lysophospholipids on leukemic cells lines. I. Differential action of two human leukemic cell line, HL60 and K562. *Blood*, **57**, 794–797.

TRAVERS, J.B., LI, Q., KNISS, D.A. & FERTEL, R.H. (1989). Identification of functional platelet-activating factor receptors in Raji lymphoblasts. *J. Immunol.*, **143**, 3708–3713.

UBERALL, F., OBERHUBER, H., MALY, K., ZAKNUN, J., DEMUTH, L. & GRUNICKE, H.H. (1991). Hexadecylphosphocholine inhibits inositol phosphate formation and protein kinase C activity. *Cancer Res.*, **51**, 807–812.

VALLARI, D.S., AUSTINHIRST, R. & SNYDER, F. (1990). Development of specific functionally active receptors for platelet-activating factor in HL-60 cells following granulocytic differentiation. *J. Biol. Chem.*, **265**, 4261–4265.

VALONE, F.H. & RUIS, N.M. (1992). Stimulation of tumour necrosis factor release by cytotoxic analogues of platelet-activating factor. *Immunol.*, **76**, 24–29.

WIGZELL, H. & RAMSTEDT, U. (1986). Natural killer cells. In *Handbook of Experimental Immunology*, Weir, D.M., Herzenberg, L.A., Blackwell, C. & Herzenberg, L.A. (eds) pp. 60.4–60.5. Blackwell Scientific Publications: Oxford.

WILLIAMS, L.T. (1989). Signal transduction by the platelet-derived growth factor receptor. *Science*, 243, 1564–1570.

WORKMAN, P. (1991). Antitumor ether lipids: endocytosis as a determinant of cellular sensitivity. *Cancer Cells*, **3**, 315–317.

WORKMAN, P., DONALDSON, J. & LOHMEYER, M. (1991). Platelet-activating factor (PAF) antagonist WEB 2086 does not modulate the cytotoxicity of PAF or antitumor alkyl lysophospholipids ET-18-O-methyl and SRI 62-834 in HL-60 promyelocytic leukaemia cells. *Biochem. Pharmacol.*, **41**, 319–322.